# Supplementary material for: Longitudinal Changes of the Ruminal Microbiota in Angus Beef Steers
Source: Animals (Basel). 2022 Apr 20;12(9):1066. doi: 10.3390/ani12091066 (PMC9102304; doi:10.3390/ani12091066)
Supplement: Supplementary file 1 [file animals-12-01066-s001.zip › animals-1649274-supplementary.pdf]

Supplementary Material

Supplemental Table S1. Composition of the diets used during the transition and finishing periods in the feedlot. The transition diet was fed for the first 21 days, and the finishing diet for the remainder of the feedlot-finishing period.

	Transition Diet	Finishing Diet
**Ingredient, % DM**		
Corn	41.12	56.20
Dried distillers grains	22.18	19.54
Corn gluten feed	-	7.08
Soybean Hulls	15.80	-
Barley Straw	6.15	4.36
Vitamin/Mineral Premix	4.47	4.76
Corn Silage	10.27	8.05
**Total**	**100.00**	**100.00**
**Nutrient, % DM**		
Dry Matter, %	62.00	62.00
NEm, Mcal/kg	2.02	2.10
NEg, Mcal/kg	1.37	1.43
Crude Protein, %	14.63	14.51
Roughage, %	16.43	12.40
Rough NDF, %	9.28	6.92
Fat, %	5.11	5.28
Calcium, %	0.75	0.70
Phosphorus, %	0.39	0.45
Potassium, %	0.90	0.71
Magnesium, %	0.22	0.21
Sulfur, %	0.25	0.26
Added Salt, %	0.21	0.22

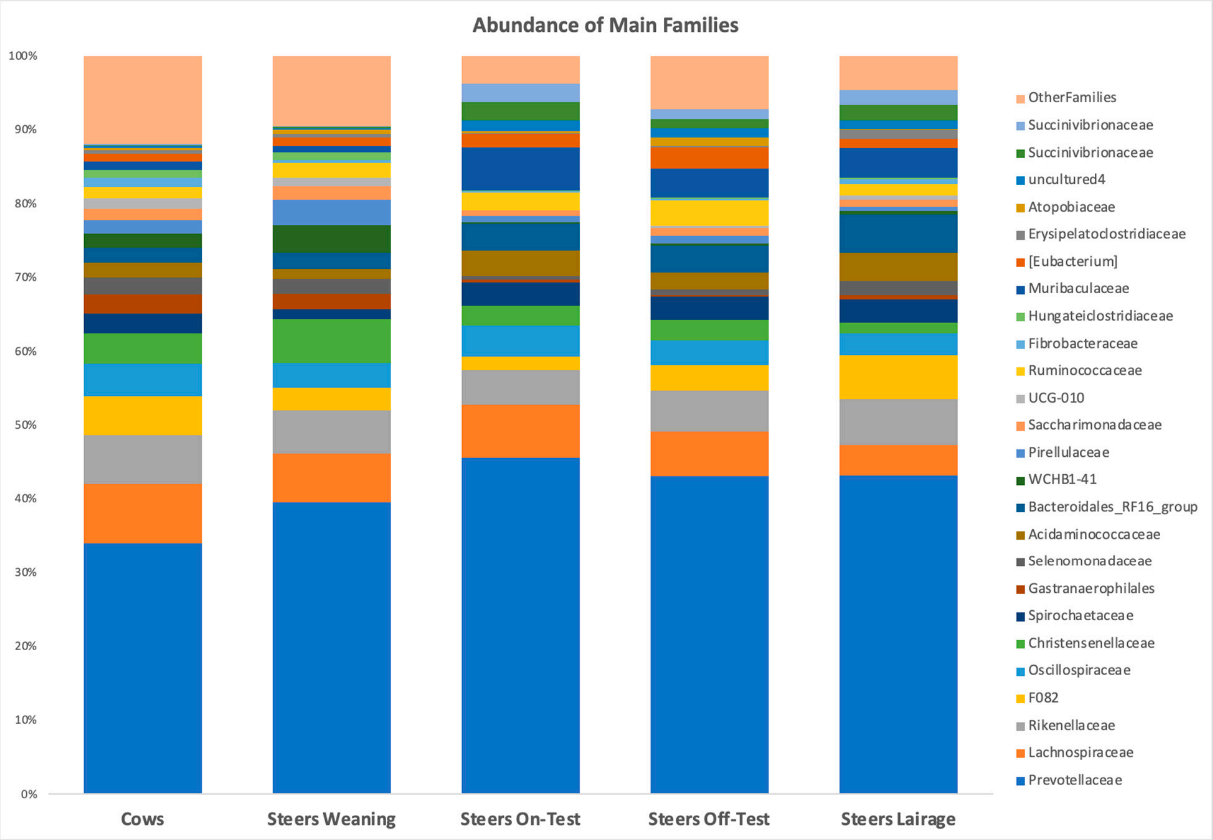

Supplemental Figure S1. Abundance of the main families in the ruminal samples of the adult cows, steers on weaning day, steers at beginning of feedlot phase (on-test), steers at end of feedlot phase (off-test), and upon arriving at the slaughterhouse (lairage).

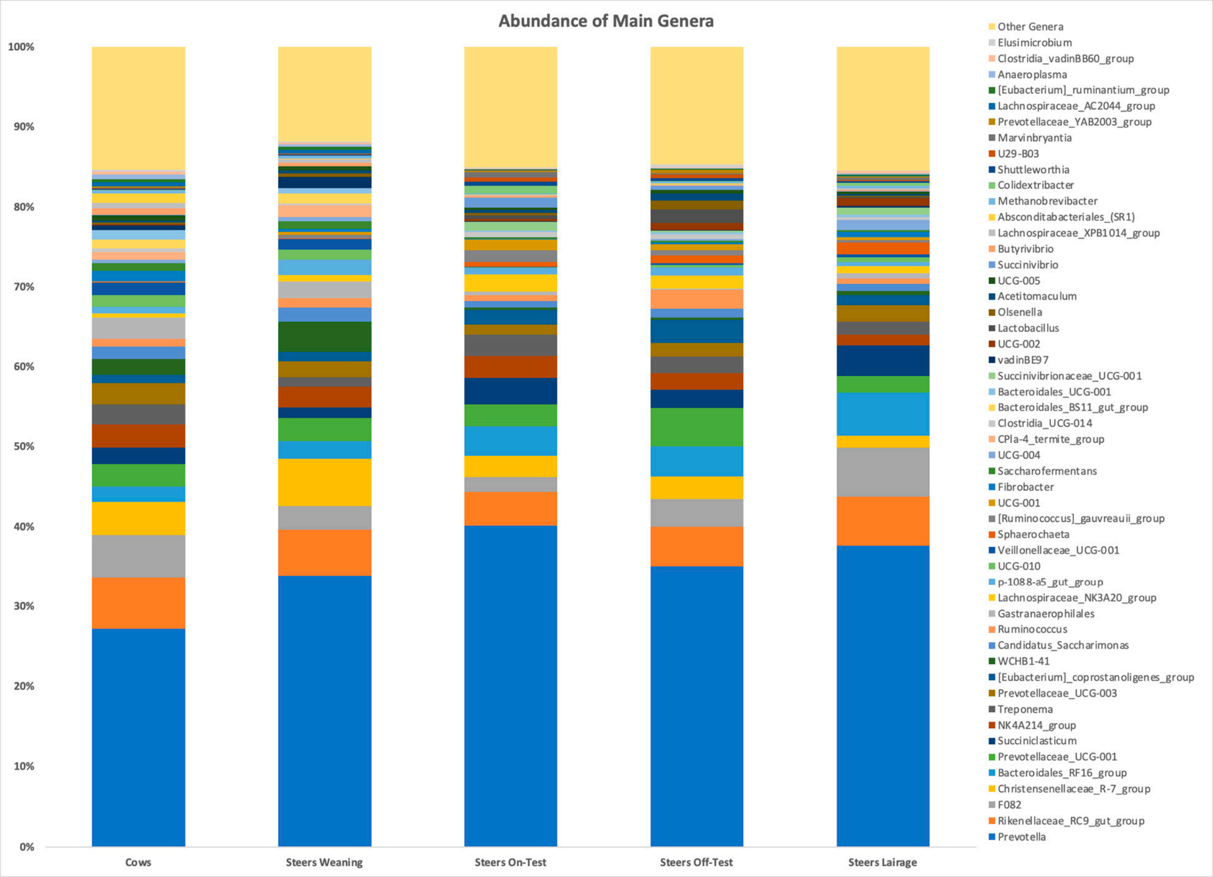

Supplemental Figure S2. Abundance of the main genera (relative abundance 0.2% or greater) in the ruminal samples of the adult cows, steers on weaning day, steers at beginning of feedlot phase (on-test), steers at end of feedlot phase (off-test), and upon arriving at the slaughterhouse (lairage).
